# Morphological Differences between Sheep and Goat Calcanea Using Two-Dimensional Geometric Morphometrics

**DOI:** 10.3390/ani12212945

**Published:** 2022-10-26

**Authors:** Lluís Lloveras, Carme Rissech, Simon Davis, Pere M. Parés-Casanova

**Affiliations:** 1 Departament d’Història i Arqueologia, Universitat de Barcelona, Montalegre 6, 08001 Barcelona, Spain; 2 Departament de Ciències Mèdiques Bàsiques, Facultat de Medicina i Ciències de la Salut, Universitat Rovira i Virgili, Sant Llorenç 21, 43201 Reus, Spain; 3 Laboratório de Arqueociências, DGPC, Calçada Do Mirante à Ajuda 10A, 1300-418 Lisbon, Portugal; 4 Escola Agrària Del Pirineu, Finca Les Colomines (Bellestar), 25711 Montferrer i Castellbò, Spain

**Keywords:** shape, *Ovis aries*, *Capra hircus*, small bovid, calcaneum, osteology, zooarchaeology

## Abstract

**Simple Summary:**

This study aims to analyse variations in the morphology of the calcaneum among sheep and goats based upon two-dimensional geometric morphometrics (GM). According to our results, clear interspecific differences in the morphology of this bone were extracted. The use of GM methods has enabled us to assess small but significant amounts of geometric variation that are difficult to measure using traditional morphometric techniques, providing a new and useful perspective to the existing literature.

**Abstract:**

The distinction between bones of sheep and bones of goats is a difficult issue in zooarchaeology. Several studies undertaken in the past to facilitate this task have relied upon both qualitative criteria and osteometry. Geometric morphometrics has proved to be a powerful tool to evaluate morphological differences in a rigorous and detailed manner. This study aims to analyse variations in the morphology of the calcaneum among sheep and goats based upon two-dimensional geometric morphometrics (GM). Twenty landmarks were selected on the surfaces of 79 calcanea (47 sheep and 32 goats) to calculate the principal components of shape variations among these specimens. Clear interspecific differences in the morphology of this bone were extracted. Most are located on the calcaneal tuber and neck, the sustentacular tali region, the articular surfaces of both the malleolus and the cubonavicular. Furthermore, the use of GM methods has enabled us to assess small but significant amounts of geometric variation that are difficult to measure using traditional morphometric techniques. They provide a new and useful perspective to what is already known in the published literature. Our results shed new light upon the possibility of the existence of qualitative features that may help to distinguish caprine breeds.

## 1. Introduction

Sheep (*Ovis aries*) and goats (*Capra hircus*) are among the first livestock species domesticated in the Fertile Crescent 10,500 years ago [1]. Since that time skeletal remains of these two taxa are generally the most common large mammals found on archaeological sites. Despite many differences between these two species, such as coat type, behaviour and temperament, the distinction between archaeological remains of sheep and goats is difficult. To deal with this issue, several studies have considered different parts of the skeleton to provide diagnostic morphological traits that help to separate the bones and teeth of sheep from those of goats. Most of these studies use qualitative criteria based on a visual morphological approach [2,3,4,5,6,7,8] while others have introduced a biometrical perspective using different indexes to solve the problem [9,10,11,12,13,14]. Osteometrical methods are commonly used to support zooarchaeological identifications made using morphological characteristics and can even be used to explore morphological variation. 

Here, we present a geometric morphometrics (GM) study of the calcaneum using newly defined landmarks. We chose the calcaneum because, like the anatomically adjacent astragalus and other tarsal bones, it is solid and compact, and hence often well preserved. However, while several studies have considered the shape of the astragalus [12,14], the calcaneum has been neglected. The GM approach, in addition to providing another identification tool for use alongside the more established morphological criteria, has the advantage of being more objective. This method considers overall shape differences, as opposed to ratio methods that analyse only parts of specimens [15,16,17].

The aims of this study are to evaluate and quantify shape and size differences between sheep and goat calcanea and to assess which morphological characters are perhaps more useful for distinguishing between these two species.

## 2. Materials and Methods

### 2.1. Study Specimens

Data were collected on 47 sheep (*Ovis aries*) and 32 goat (*Capra hircus*) calcanea (Table 1) in the reference collection of the Laboratório de Arqueociências (Direção Geral do Património Cultural, Lisbon, Portugal) and the National Museum of Natural History and Science (Lisbon, Portugal). All calcanea belong to domesticated specimens. Generally, the right calcaneum was studied, however, in the case of a missing, broken, or pathological right calcaneum the left one was considered instead. In these cases, a reflection of the obtained images was used to obtain the shape of a right-oriented calcaneum. Only adult and subadult specimens were included in this study, determined by having the calcaneal tuber completely fused or fused but with the epiphyseal line visible. Specimens were sampled by taxa and evenly across breeds. Pathological specimens were not studied.

### 2.2. Geometric Morphometrics

Here a two-dimensional (2D) geometric morphometric approach is used to quantify, analyse, and visualize morphological variation. Images were taken perpendicular to the medial side of each calcaneum from a distance of 30 cm with a Nikon^®^ D3400 digital camera on a fixed position equipped with an 18–55 mm objective set at a focal distance of 55 mm. Images were saved in JPG format. 

**Figure 1:**
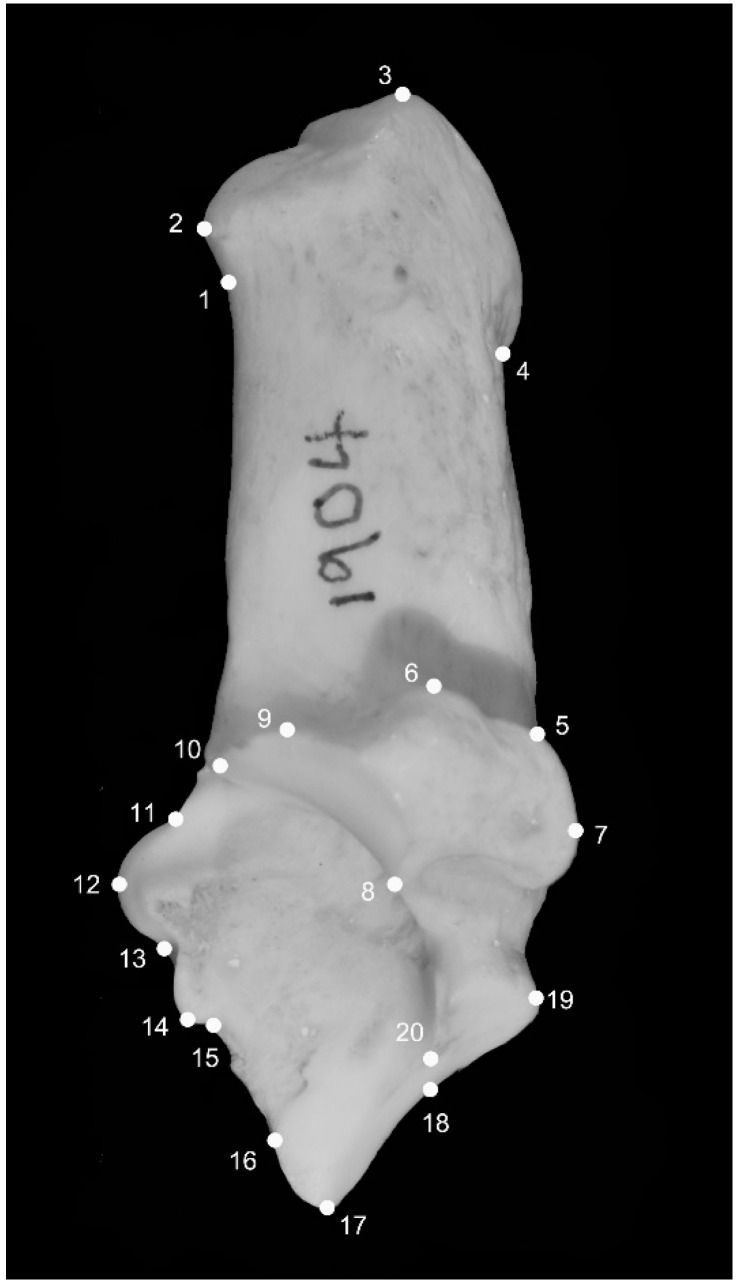
Landmark locations on the medial view of the sheep and goat calcanea. For the definition of each landmark, see the text.

2D GM analyses morphological features by collecting a series of coordinate data (x, y) across a feature of interest. Landmarks, which are anatomically specific loci [18,19] were collected using the TPS series (TPSutil and TPSdig) of software [20]. A total of twenty landmarks were selected on the calcaneum (Figure 1). These are the definitions of the landmarks (see also Figure 1): (1) Most concave point below the calcaneal tuber; (2) Anterosuperior point of the calcaneal tuber; (3) Most superior point of the calcaneal tuber; (4) Posteroinferior point of gastrocnemius enthesis; (5) Intersection point between the calcaneum neck and sustentacular tali; (6) Uppermost point of sustentacular tali; (7) Most posterior point of sustentacular tali; (8) Most anteroinferior point of sustentacular tali; (9) Uppermost point of superior astragalus articular surface; (10) Most anterior point of superior astragalus articular surface; (11) Most concave point between landmarks 10 and 12; (12) Most anterior point of malleolus articular surface; (13) Most concave point between landmarks 12 and 14; (14) Most inferior point of malleolus articular surface; (15) Most concave point inferior to the malleolus articular surface; (16) Most anteroinferior point of inferior astragalus articular surface; (17) Most anteroinferior point of cubonavicular articular surface; (18) Most concave point of the posterior edge of the cubonavicular articular surface; (19) Most posterior point of cubonavicular articular surface; (20) Most concave point of the anterior edge of the cubonavicular articular surface.

Before proceeding further with the data analyses, we conducted a repeatability experiment to quantify photographic and digitising errors on the current set of landmarks to be sure that the methods used for quantifying calcanea shape differences were reliable and consistent. First, we repeated (the first author) the photographs of the same specimen ten times during ten days, mounting and dismounting both camera and tripod each time. In addition to these photographs, those of ten different specimens were randomly chosen. Later, each landmark was located on the twenty photographs twice during two weeks. A Procrustes ANOVA comparing variation in landmark location both among and within calcanea was performed [21]. 

To determine shape differences between sheep and goat calcanea, coordinate data resulting from the current set of landmarks were projected into a common shape space using a generalized Procrustes analysis (GPA). The GPA relocates configurations to a common origin, scales them to centroid size, and rotates them producing a new set of shape variables, Procrustes coordinates, that can then be analysed using standard multivariate methods [16,22]. Data were further analysed for outliers, a covariance matrix was generated from this dataset, and a principal component analysis (PCA) was conducted on this covariance matrix. A PCA is an ordination method used to reduce the dimensionality of the data and to display variation on uncorrelated principal components. This method has proved to work very well as an exploratory tool to investigate morphological heterogeneity [16,23].

To assess the influence of allometry on the data, a multivariate regression was conducted using the centroid size as the independent variable and the calcanea shape (Procrustes coordinates) as the dependent variable.

We are aware that the sample is small, and results should be taken with caution, but in order to assess possible differences in shape among different breeds, the category ‘breed’ was also included as a classifier. A canonical analysis of variance (CVA) was performed to maximise variation between groups relative to within-group variation and for confirming group membership [16].

All analyses were executed in MorphoJ v1.06d [15].

## 3. Results

The Procrustes ANOVA showed highly significant differences between calcanea, both in size and shape (*p* < 0.0001). The mean squares for calcanea size variation (MS calcanea = 34,702.87) exceeded the mean squares for the replicates (MS error = 82.09) by a factor of 34,620.78. For shape, mean squares for calcanea variation (MS calcanea = 0.0001820118) exceeded the mean squares for the replicates (MS error = 0.0000080207) by a factor of 0.0001739911. These results indicate low measurement error and consequently strong repeatability of the landmark location on the calcanea.

The multivariate regression of shape on centroid size indicated a low allometric effect (4.5%) in the data but it was significant (*p* = 0.0021) based on a permutation 10,000 rounds. To remove the allometric effect, a PCA was run on a covariance matrix of residuals from the multivariate regression. 

**Figure 2:**
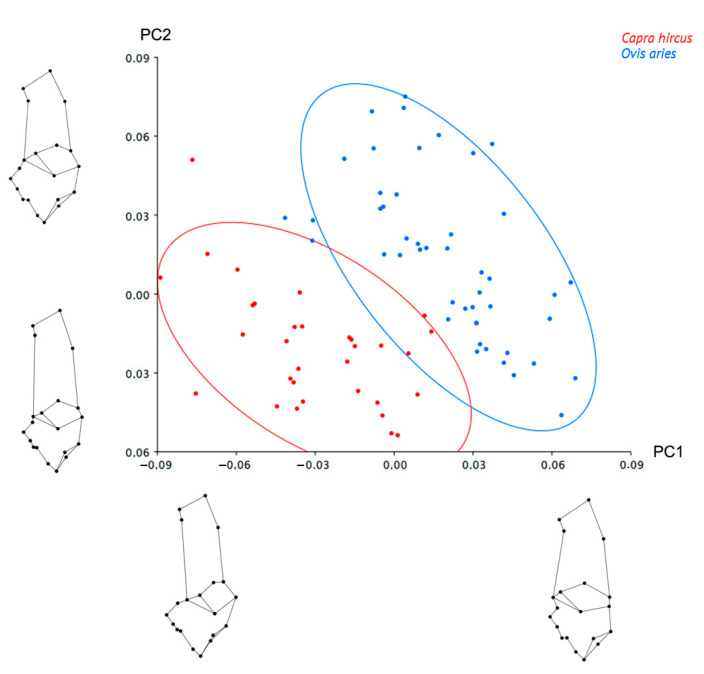
Scatterplot of the principal component analysis of sheep (*Ovis aries*) and goats (*Capra hircus)* based on the weight matrix of residuals from the multivariate regression with the corresponding extreme calcanea shapes of every axis. PC1: 30.9% of the variance, PC2: 22.9% of the variance. C (red): goats, O (blue): sheep. 90% confidence ellipse.

In the PCA, the first two principal components accounted for 53.8% of the cumulative shape variation. The first principal component (PC1) explained 30.9% and the second (PC2) 22.9% of the variation. Distribution of the different individuals in the shape space defined by these components showed that calcanea shape varied according to taxa along both the first and second principal components (Figure 2). Along PC1, the traits that accounted for this variation were: an increase in the anterior concavity below the calcaneal tuber; a width increase of the calcaneum neck, particularly in the region close to the sustentacular tali and the superior astragalus articular surface; the region that encompasses the sustentacular tali becomes wider and less posteriorly prominent; the region of the malleolus articular surface becomes less anteriorly prominent; and the cubonavicular articular surface increases. Along PC2, the traits that accounted for variation were descending from the maximum concavity point and a concavity increase both bellow the anterior calcaneal tuber; an increase of the superior astragalus articular surface; an enlargement of the region encompassing the malleolus articular surface; and a shortening of the region between the most concave point inferior to the malleolus articular surface and the most anteroinferior point of the cubonavicular articular surface (Figure 2).

**Figure 3:**
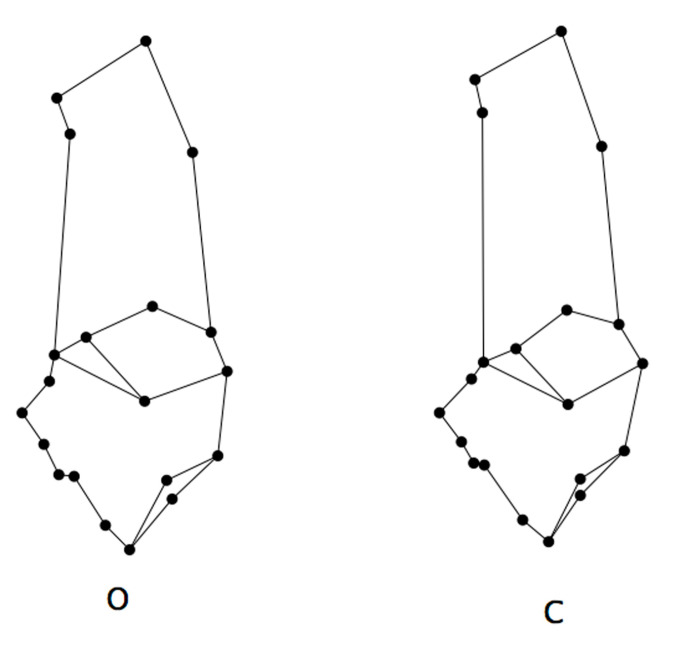
Calcanea average shape for sheep (**O**) and goat (**C**).

In general terms (Figure 3), in sheep the calcaneal tuber tends to be more concave in the anterior side and the calcaneum neck and the sustentacular tali region are wider. In goats, the malleolus articular surface region is shorter and more prominent anteriorly. The cubonavicular articular surface is wider in sheep.

**Figure 4:**
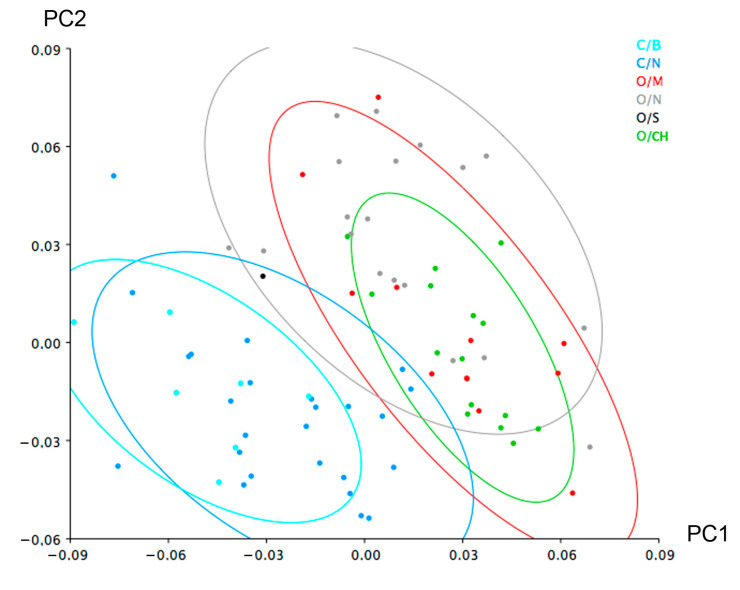
Scatterplot of the principal component analysis of sheep and goats (see Figure 2) with the data about the different breeds. O: sheep, C: goat, M: merina, S: soay, CH: churra, B: balearic, N: non-determined breed. 90% confidence ellipse.

**Figure 5:**
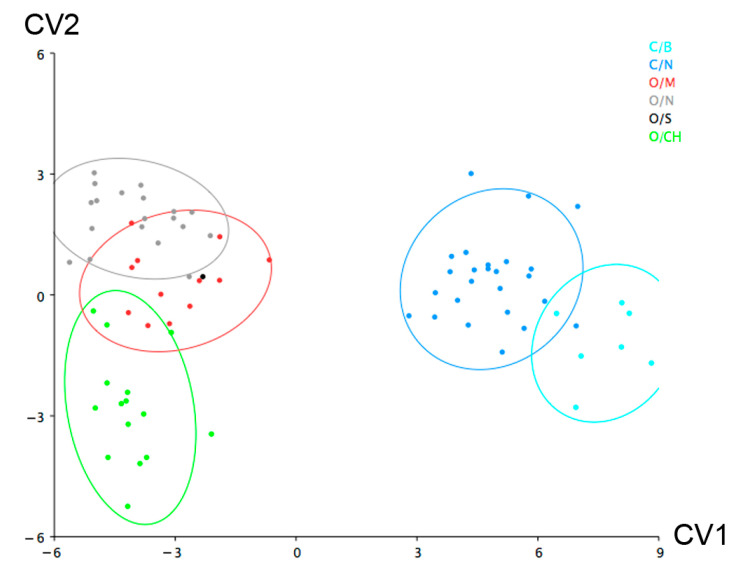
Canonical variate analysis of the different breeds by form in sheep and boat calcanea. O: sheep, C: goat, M: merina, S: soay, CH: churra, B: balearic, N: non-determined breed. 90% confidence ellipse.

When the category ‘breed’ was also included as a classifier in the PCA (Figure 4), within sheep taxa, despite the overlap, a tendency to cluster is observed. To evaluate this tendency and identify the variation among the groups, a canonical variate analysis (CVA) was also conducted on the residuals of the multivariate regression (Figure 5). A permutation test with 10,000 permutations based upon the Mahalanobis distances between groups returned significant probabilities (Table 2) indicating shape variation among sheep breeds, particularly Churras (CH) and Merinas (M). The only non-significant results are those involving the Soay breed, which is due to the fact that it is represented by a single individual (Table 1).

## 4. Discussion

GM and associated statistical analyses have allowed us to describe the morphological differences among sheep and goat calcanea. Results revealed that calcaneum shape varies significantly between these two species whilst the size differences proved worthless after removing the allometric effect. According to our results shape differences are mostly located on the calcaneal tuber and neck, the sustentacular tali region, the malleolus articular surface and the cubonavicular articular surface.

Differences in the concavity of the anterior surface of the calcaneal tuber and the widening of the calcaneum neck agree with the qualitative analysis conducted by Prummel and Frisch [5] and Clutton-Brock et al. [24] who observed that the *corpus calcanei* is more or less concave in sheep whilst in goats it is straight to convex. 

The widening of the calcaneum neck in sheep also results in a broadening of the sustentacular tali region. In fact, differences are in the metrical relationship between the height and the width of this region. The ratio between the distance landmarks 6–8 (height of sustentacular tali) and the distance landmarks 7–9 (width of the sustentacular tali) is higher in goats ($\bar{x}$ = 0.74, DS = 0.085) than in sheep ($\bar{x}$ = 0.65, DS = 0.082). These differences are statistically significant (Student’s t = −3.313, *p* = 0.002*). This is consistent with the results obtained by Salvagno and Albarella [14] who reported that there is a significant difference in the depth of the sustentacular tali to the length of the malleolus articular surface ratio in sheep in relation to goats. In addition, when the distance between landmarks 8 and 9 is compared, sheep show higher values than goats (sheep: $\bar{x}$ = 1.06, DS = 0.12; goats: $\bar{x}$ = 0.96, DS = 0.09; Student’s t = 2.99, *p* = 0.005). Since this distance is related to the depth of the superior astragalus articular surface, our results reinforce the observations of Salvagno and Albarella [14]. All these features indicate a great robustness of this entire region in sheep calcanea. 

According to our results, the malleolus articular surface is more elongated in sheep than in goats. This morphological difference is consistent with the observations of Boessneck [2] who, using qualitative criteria, suggested that the length of the articular facet for the os malleolare on the lateral process is greater than half of the entire process in sheep, while in goat it is smaller. This variation makes this a region of particular interest to separate both taxa, providing also good results when applying ratios that use the length of the articular facet [13].

In goats, the cubonavicular articular surface tends to be more concave in the anterior side (Figure 2 and Figure 3) making the articular surface wider in sheep. Differences among sheep and goats in this anatomical region have not been explored, however researchers have found that the length of the cubonavicular facet is significantly longer in open-adapted bovids than in bovids from forested environments. The cubonavicular facet is also significantly longer in wild cursorial bovids than in semi-free and domestic cursorial bovids, like sheep [25,26]. All these results point to a functional signal in these differences. In fact, the morphology of the calcaneum is considered to clearly reflect lifestyle-related functional adaptation in artiodactyls [26]. Similarly, using GM analysis, sheep astragali have been found to have flatter and elongated plantar surfaces and to be more compressed than those of sheep [14]. Thus, the morphological differences registered in this study might be due to the different locomotor behaviours of sheep and goats. While the sheep is more robust and able to walk long distances across flat land and open country in search of food [27], the goat is more agile and adapted to forest and used to negotiating rocky escarpments. The calcaneum is the main region of insertion for the plantar flexor muscles of the ankle which emphasizes the important functional role of this bone in the plantar flexion of the ankle. In fact, a shorter calcaneum increases speed in plantar flexion, which is advantageous in open-country animals such as sheep during their rapid flight across level ground. A longer calcaneum increases power in plantar flexion, which is favoured in forest animals such as the goat with more complicated locomotor behaviours that include leaping over downed trees and for negotiating complex forest paths and rocky cliffs [25].

Finally, our results have also indicated the potential of GM analysis to distinguish different breeds of sheep. This is of particular interest as studies conducted in sheep and goat astragali have shown the difficulty in separating different breeds using metrical methods [12]. However, a GM study using larger samples of known breeds is needed to confirm our observations. 

## 5. Conclusions

The findings in this study constitute a first approach towards elucidating shape differences between sheep and goat calcanea by using GM of landmark-based data. The results obtained agree with the morphological patterns of sheep and goat calcanea described in previous studies. The use of GM methods has enabled us to assess small but significant amounts of geometric variation that are difficult to measure using traditional morphometric techniques, providing a new and useful perspective to the existing literature. 

Our results also shed new light on the possible existence of qualitative features that may help to distinguish breeds of sheep. However, more research is needed using larger samples to confirm and explain these findings.

According to our results the shape differences across sheep and goat calcanea are mostly located on the calcaneal tuber and neck, the sustentacular tali region, the malleolus articular surface and the cubonavicular articular surface. In general terms, in sheep, the calcaneal tuber tends to be more concave on the anterior side and the calcaneum neck, the sustentacular tali region and the cubonavicular articular surface tend to be wider. In opposition, in goats, the malleolus articular surface region tends to be shorter and more prominent anteriorly. These morphological differences could reflect the functional adaptation of these animals in different habitats that demand different methods of locomotion. While the sheep is adapted to running across flat land, the goat is adapted to rocky escarpments. 

These results reveal that GM analyses can be very useful to understand differences between sheep and goat bones.

## Figures and Tables

**Table 1:** Number and breed (or origin) of the specimens of sheep and goat included in this study.

Sheep (*Ovis aries*)	N
churra	15
merina branca	2
merina preta	4
merina	7
soay	1
unknown	18
Total	47
Goat (*Capra hircus*)	
balearic	7
unknown	25
Total	32

**Table 2:** Mahalanobis distances among groups. * Significant *p*-values from permutation tests (10,000 permutation rounds). O: sheep, C: goat, M: merina, S: soay, CH: churra, B: balearic, N: non-determined breed.

	C, B	C, N	O, M	O, N	O, S
C, N	60,177 *				
O, M	114,638 *	84,345 *			
O, N	124,554 *	91,746 *	36,790 *		
O, S	129,373	100,329	70,437	76,682	
O, CH	126,275 *	95,637 *	45,164 *	48,286 *	80,174

## Data Availability

The data presented in this study are available on request from the corresponding author.
